# Genetic risk in telomere biology disorders: it adds up

**DOI:** 10.1172/JCI195921

**Published:** 2025-08-15

**Authors:** Tanner O. Monroe

**Affiliations:** Center for Genetic Medicine, Feinberg School of Medicine, Northwestern University, Chicago, Illinois, USA.

## Abstract

For many conditions, genotyping aids in clinical decision making. However, interpreting the clinical significance of genetic variants remains challenging, in part because a single risk variant does not always lead to disease, and variant carriers experience variable outcomes. One hypothesis underlying these phenomena, which are known as *incomplete penetrance* and *variable expressivity*, respectively, is that additional common genetic variation beyond the primary variant influences the presence and severity of disease. In this issue of *JCI*, Poeschla et al. present a compelling argument that common variants linked to telomere length act together with high-risk telomere biology disorder variants to scale outcomes. These data support a model in which many variants interact to shape cumulative risk.

## An evolving perspective of disease heritability

Before completion of the Human Genome Project, high-effect risk variants were discovered and annotated using linkage analysis and positional cloning, starting from large pedigrees of affected and unaffected relatives (1). Discovery of a “disease gene” then enabled extended family cascade genetic testing to help identify individuals at risk. Physicians now routinely use panel sequencing, which covers many specific genes, to screen individuals showing symptoms consistent with heritable disease. These panels can sometimes provide actionable results when pathogenic variants are discovered. However, panels often either fail to detect a pathogenic variant or they reveal variants of uncertain significance (VUS), results that are discouraging to both clinicians and patients (2). As genome-wide association studies (GWAS) gained momentum in the 2010s, it started to become clear that common, often noncoding, variants, which individually confer minimal risk, can accumulate and additively increase genetic liability. Most people carry many such variants. The cumulative burden of those variants can be quantified as polygenic scores (PGS), in which an individual’s risk variants are noted, scaled by effect size, and added together (2). A PGS is therefore a simple metric that accounts for many variants thought to contribute to a trait in any individual. The phenotypic impact of a statistically extreme PGS can often be comparable to that of a single, large effect variant detectible on a clinical sequencing panel (3). The Electronic Medical Records and Genomics (eMERGE) Network has even begun evaluating the efficacy of returning these relatively complicated genetic results to patients (4). By combining these 2 approaches, investigators at the forefront of this technology are now finding that common, low-effect variants accounted for in an individual’s PGS can modify the penetrance and expressivity of rare, high-risk panel variants (4, 5). One can envision a future where genetic risk is communicated not as presence/absence, but as a scale relative to population average.

## Common and rare variation in telomere biology disorders

**Figure 1:**
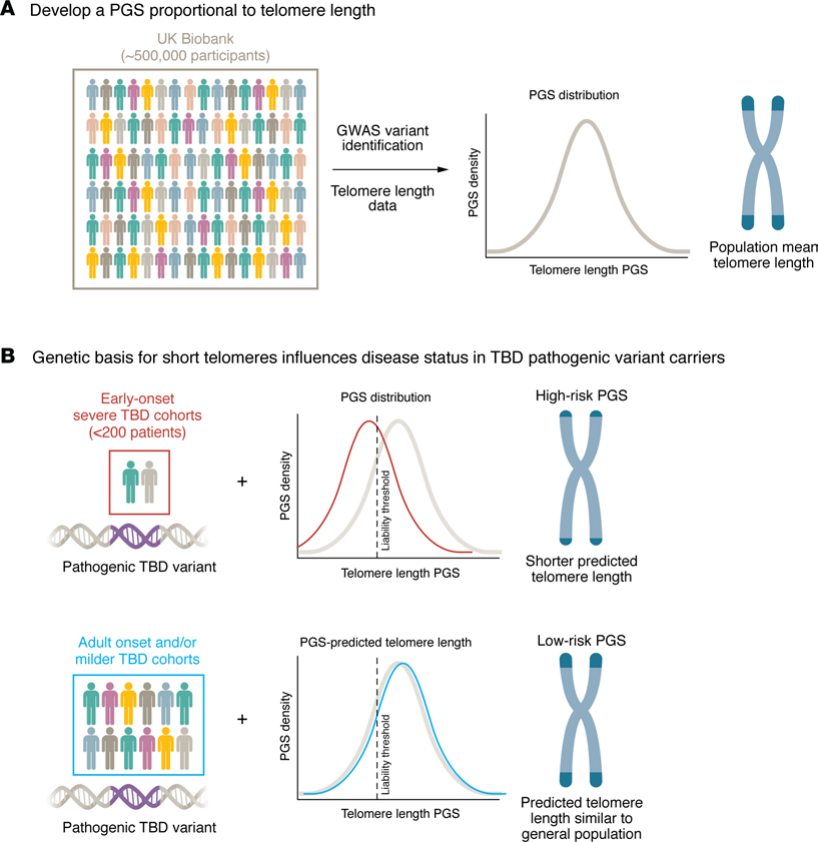
Polygenic background modifies disease probability in carriers of rare pathogenic telomere biology disorder (TBD) variants. Rare telomere biology disorder (TBD) pathogenic genetic variants do not always result in overt disease, and affected individuals exhibit considerable phenotypic variability — from relatively minor to severe. One hypothesis is that background genetic variation beyond the primary pathogenic variant contributes to this variety of outcomes. **(A)** Common variants associated with mean telomere length through a GWAS performed by Poeschla and colleagues can be used to generate polygenic scores (PGS) that represent telomere length predisposition. **(B)** They found that this score shifts the total genetic liability in individuals who also carry rare TBD pathogenic variants, influencing both the probability of developing TBD and disease severity. Individuals with a a high-risk PGS (short telomere predisposition; top) have increased risk of crossing a TBD liability threshold, often manifesting as early onset disease, while those with a low-risk PGS (long telomere predisposition; bottom) are less likely to manifest disease, despite carrying the same rare variant. Importantly, the genetic liability threshold for TBD is only relevant in the context a pathogenic variant, and there are some individuals to the left of the liability threshold who do not have severe disease, indicating the complex genetic architecture yet to be uncovered.

In this issue of the *JCI*, Poeschla and colleagues (6) add to the rapidly growing body of literature that considers the broader genomic context for variant risk stratification. Their study focuses on patients with telomere biology disorders (TBDs), a group of extremely rare and remarkably heterogeneous diseases thought to be driven by pathologic germline variants affecting telomere maintenance. To understand how common genetic variation impacts the penetrance and expressivity of variants thought to cause TBDs, Poeschla et al. developed a PGS based on common SNPs across the genome associated with telomere length in UK Biobank participants. Using two independent biobanks of young individuals diagnosed with severe TBD, they report that, in carriers of rare, high-risk TBD variants, the distribution of PGS-predicted telomere length skews smaller than that of the reference population in the UK Biobank. Additionally, the UK Biobank also includes carriers of rare, high-risk TBD variants who did not manifest severe early onset disease. In contrast to the early onset TBD cases, the rare, high-risk TBD variant carriers in the UK Biobank had a PGS distribution resembling the general population. These findings suggest that the combination of a high-risk variant plus background genetic risk for short telomeres increases the risk of severe childhood TBD (Figure 1).

While elegant and intuitive as a first step, it is clear this approach still misses components of the complete TBD genetic architecture as well as “gene × environment” interactions. Notably, despite phenotypic differences at the cohort level, there remains considerable overlap between the PGS distributions in rare, high-risk TBD variant carriers who experience childhood disease and those who do not. The authors found that, among UK Biobank carriers of rare, high-risk TBD variants, incidence of adult-onset TBD, in the form of idiopathic pulmonary fibrosi, was higher in those with PGS indicative of short telomeres compared with those with a PGS for long telomeres. Given the biobank data from young individuals diagnosed with severe TBD, one wonders why these adults with pulmonary fibrosis who carry both a high effect variant and short telomere PGS do not display more severe and earlier diagnosed disease. This transition from population genetics to personalized medicine is among the most sizable barriers to clinical implementation of genomic insights. Despite these unresolved nuances, the population level results still point toward a future where a combined analysis of common and rare variants meaningfully estimates outcomes.

## Accessible phenotype proxies can help resolve rare disorders

Generally, GWAS are not performed for very rare conditions because it is challenging to assemble large cohorts of individuals affected by the disease. TBDs are no exception, with an approximate prevalence close to one in a million individuals diagnosed with the archetypal TBD, dyskeratosis congenita. Therefore, to quantify background genetic variation that might influence TBD, Poeschla and colleagues cleverly performed a GWAS on a proxy phenotype — mean telomere length — which can be assessed in datasets from the UK Biobank. Mean telomere length does not reflect the underlying disease etiology for TBD, which is attributed to the shortest telomeres (7). Still, the authors surmised that the underlying biology might be related, and their hypothesis was supported in this study by variant prioritization of the UK Biobank GWAS signals converged on cellular processes and genes known to contribute to TBD. Given that telomere length in any individual varies by age and tissue source (8), it is likely that performing the GWAS on a trait more directly related to the cellular pathomechanism underlying TBD would reveal information closer to the ground truth. Unfortunately, data on those traits may never be available at appropriate scale. Nonetheless, the approach taken by Poeschla and colleagues suggests that incorporating genetic signals for biologically related, accessible traits can serve as a practical approach for other rare disease researchers lacking access to nonstandard phenotype information. For example, a nephrologist might consider incorporating blood pressure PGS when assessing rare kidney disease gene penetrance.

## Future directions and clinical implications

Aside from relatively few conditions, predicting outcomes based on gene variant carrier status remains extremely challenging. This complexity has become more apparent with the development of large biobanks in which investigators have identified relatively unaffected individuals carrying annotated pathogenic variants (9, 10). Even the textbook inheritance of pea plant traits has proven more nuanced than Mendelianists, and perhaps Gregor Mendel himself, liked to admit (11, 12). The recent availability of deeply sequenced cohorts linked to electronic health records is helping researchers resolve the complexity of underlying traits that were once considered genetically simple by enabling more comprehensive detection of genetic signals and more precise phenotyping.

Sixty years ago, D.S. Falconer proposed a liability threshold model of disease wherein low effect variants can accumulate to total genetic liability for common disease (13). Later, he proposed that common variants might also contribute to rare disease in combination with medium and large effect variants. It is becoming realistic to move from Falconer’s theory to clinical applications (14). Notably, Falconer’s model is a simple variant additivity approach. Epistasis, where the effect of one variant is masked or amplified in the context of another, demonstrably exists and can result in nonlinearities in genotype-phenotype correlations (15), but theoretical and empirical data suggest that such cases are the exception (16). Therefore, simple additive models may be realistically actionable in the relatively near term. Therefore, clinicians should be prepared to anticipate the implementation of clinical genomic risk tests that account for many layers of genetic information across the allele frequency and functional genomics spectrum. These tests will convey genetic risk not as presence/absence, but as a continuum of liability.

Even after accounting for all genetic risk, total phenotype variation in the population is a function of both genes and the environment (17). It is unclear at what point we will reach an upper bound on genetic prognoses, but we are not there yet. Meanwhile, this work from Poeschla et al. represents an important intermediate step towards the next phase of clinical genotyping.

## Version 1 08/15/2025

Electronic publication

## References

[1] No authors listed (1993). A novel gene containing a trinucleotide repeat that is expanded and unstable on Huntington’s disease chromosomes. A novel gene containing a trinucleotide repeat that is expanded and unstable on Huntington’s disease chromosomes. The Huntington’s Disease Collaborative Research Group. Cell.

[2] Mighton C (2021). Clinical and psychological outcomes of receiving a variant of uncertain significance from multigene panel testing or genomic sequencing: a systematic review and meta-analysis. Genet Med.

[3] Khera AV (2018). Genome-wide polygenic scores for common diseases identify individuals with risk equivalent to monogenic mutations. Nat Genet.

[4] Lennon NJ (2024). Selection, optimization and validation of ten chronic disease polygenic risk scores for clinical implementation in diverse US populations. Nat Med.

[5] Niemi MEK (2018). Common genetic variants contribute to risk of rare severe neurodevelopmental disorders. Nature.

[6] Poeschla M (2025). Polygenic modifiers impact penetrance and expressivity in telomere biology disorders. J Clin Invest.

[7] Hemann MT (2001). The shortest telomere, not average telomere length, is critical for cell viability and chromosome stability. Cell.

[8] Demanelis K (2020). Determinants of telomere length across human tissues. Science.

[9] McGurk KA (2023). The penetrance of rare variants in cardiomyopathy-associated genes: A cross-sectional approach to estimating penetrance for secondary findings. Am J Hum Genet.

[10] Wright CF (2024). Guidance for estimating penetrance of monogenic disease-causing variants in population cohorts. Nat Genet.

[11] Feng C (2025). Genomic and genetic insights into Mendel’s pea genes. Nature.

[12] Radick G (2024). Alternative paths for genetics, then and now: Q&A with Gregory Radick about Disputed Inheritance. Trends Genet.

[13] Falconer DS (1965). The inheritance of liability to certain diseases estimated from incidence among relatives. Ann Hum Genet.

[14] Kingdom R (2024). Genetic modifiers of rare variants in monogenic developmental disorder loci. Nat Genet.

[15] Domingo J (2019). The causes and consequences of genetic interactions (epistasis). Annu Rev Genomics Hum Genet.

[16] Hivert V (2021). Estimation of non-additive genetic variance in human complex traits from a large sample of unrelated individuals. Am J Hum Genet.

[17] Brandes N (2022). Open problems in human trait genetics. Genome Biol.

